# Assessment of post-partum physical exercise practice and its associated factors among women in postpartum period, in West Wollega zone, Oromia, Ethiopia

**DOI:** 10.3389/fpubh.2025.1505303

**Published:** 2025-01-23

**Authors:** Tegegne Yadeta, Daniel Belema, Seifedin Ahmad, Nurye Sirage, Abubeker Seid Ali, Kedir Ali, Ali Yimer

**Affiliations:** ^1^Department of Nursing, Nedjo Health Center, Nedjo, Ethiopia; ^2^Department of Midwifery, College of Health Sciences and Referral Hospital, Ambo University, Ambo, Ethiopia; ^3^Department of Public Health, College of Health Sciences and Referral Hospital, Ambo University, Ambo, Ethiopia; ^4^Department of Midwifery, College of Health Sciences, Woldia University, Woldia, Ethiopia; ^5^Department of Nursing, College of Health Sciences, Woldia University, Woldia, Ethiopia; ^6^Department of Public Health, College of Health Sciences, Woldia University, Woldia, Ethiopia

**Keywords:** postpartum physical exercise, postpartum women, Ethiopia, physical exercise, postpartum period

## Abstract

**Background:**

The postnatal period, spanning the initial 6 weeks after childbirth, brings about a range of difficulties for mothers globally, encompassing psychological, physiological, and biological shifts. Incorporating regular physical activity during this phase has demonstrated the potential to mitigate the chances of chronic metabolic disorders. Nevertheless, there is a lack of comprehensive understanding regarding women’s engagement in postpartum physical exercise during this time.

**Methods:**

The research employed a community-based cross-sectional study design carried out between April 20 and May 20, 2023. A sample of 422 women was chosen through a simple random sampling method utilizing computer-generated random numbers. The data was inputted into Epi-Data 4.6 and processed with SPSS 26. Descriptive statistics and logistic regression were employed for the analysis. Variables with a *p*-value below 0.25 in the bi-variable analysis were included in the multivariable analysis, and those with a *p*-value under 0.05 in the multivariable analysis were considered as significantly associated factors. The results were presented using graphs and tables.

**Result:**

This research involved 416 women, achieving a response rate of 98.6%. The prevalence of postpartum physical exercise practice among the participants was determined to be 19.5% (95% CI: 12.03–22.50). Several factors were independently associated with postpartum physical exercise practice: Women with a college education or higher had nearly three times greater odds of engaging in postpartum physical exercise (AOR = 2.97, 95% CI: 1.14, 7.80). Being employed in a government institution was associated with a 2.14 times higher likelihood of participating in postpartum physical exercise (AOR = 2.14; 95% CI: 1.96, 4.74). Primipara women had 2.8 times higher odds of practicing postpartum physical exercise (AOR = 2.80, 95% CI: 1.72, 4.59). Women who had exercised before pregnancy were more likely to continue postpartum physical exercise, with 5.1 times higher odds (AOR = 5.10; 95% CI: 2.78, 9.29). Possessing good knowledge about postpartum physical exercise was associated with 2.3 times greater odds of engaging in such exercise (AOR = 2.30; 95% CI: 1.20, 4.40).

**Conclusion:**

The study indicates that the majority of participants were not participating in postpartum physical exercise. Nevertheless, women with higher levels of education, government jobs, primipara mothers, prior exercise experience before pregnancy, and a good knowledge of postpartum physical exercise were more inclined to engage in it. These results underscore the need to increase awareness among healthcare providers and other relevant organizations about the advantages of postpartum physical exercise in preventing complications during the postpartum phase.

## Introduction

Postpartum physical exercise refers to engaging in workout routines during the 6 weeks following childbirth. These exercises help ensure optimal functioning of various bodily systems and reduce the risk of complications (1). Engaging in exercise after giving birth can accelerate the healing process, promote the development of essential strength, and help alleviate back discomfort. By incorporating postpartum exercise into their routine, women can aid their recovery and enhance their physical well-being after childbirth (2). Women are particularly vulnerable to various challenges during the postpartum period, including exhaustion, sleep disturbances, anxiety, depression, sexual issues, physical limitations, and dissatisfaction with partner support. These issues can significantly impact their overall quality of life, emphasizing the importance of providing adequate support and resources to address these postpartum concerns (3). The postpartum period can be a highly stressful time for women worldwide, as it involves significant life, physical, and emotional changes. Additionally, women commonly face various challenges during this period, including issues like uterine prolapse, the risk of non-communicable diseases, and weight gain (4). These factors contribute to the complexity and importance of providing adequate support and care for women during the postpartum phase. They are encouraged to start and maintain moderate to intensity physical exercise using both aerobic and muscle conditioning exercises during the postpartum period in the absence of medical or obstetrical complications under the guidance of health care Providers (5). The benefits of physical exercise for women include improved aerobic fitness, decreased body fat, improved bone mineralization, decreased depression, and a decreased risk of colon cancer, hypertension, type 2 diabetes, and osteoporosis fracture (6).

In the United States, studies have shown that postpartum fatigue affects a significant percentage of mothers, ranging from 44 to 95%. This fatigue typically begins shortly after giving birth and reaches its peak severity within 36 h (7, 8). Postpartum physical exercises, such as Kegel’s, abdominal, and breathing exercises, play a crucial role in improving pelvic floor and abdominal muscle tone, bowel and bladder function, as well as cardiovascular fitness. These exercises can be beneficial for postpartum women in regaining strength and promoting overall physical well-being (9).

Postpartum physical exercise has become a fundamental aspect of women’s lives and an important constituent of postnatal care (10). American College of Obstetrician and Gynecology recommended that low to a moderate regular impact exercise regime for postnatal mothers performed 3 times per week for at least 20 to 30 min and gradually progressed over a period of time can be followed to improve overall fitness (11). The study in Uganda shows that inadequate knowledge among postpartum women concludes that the health awareness program on postpartum physical exercises should be informed by the health personnel to improve the knowledge practices of postpartum exercise among women (12).

Some of the irrational beliefs and customs in society prohibit women from practicing postpartum physical exercises; hence women with newborn babies encounter many social barriers in most areas of African countries (12, 13). To create a culture of adherence among all postnatal mothers, this study is intended to assess the practice of post-partum physical exercise among postpartum women.

In the general population, Physical inactivity is the fourth leading risk factor of mortality worldwide (14). Global assessments of impact due to low knowledge and practices postpartum exercise among postpartum women were shown, with estimates of 5–21% and 15–45% in developed and developing countries, respectively (15). Unsurprisingly, physical inactivity causes one in six maternal morbidity in the UK and is expected to cost the country £7.4 billion yearly (16). In the UK, 42% of women are estimated to be not active enough to maintain their postpartum exercise (17). The incidence and prevalence rates of postpartum mothers developing health complications during the postpartum period are also high in developing nations compared to developed nations (8).

However, the evidence that is currently available shows that women who participate in vigorous postpartum exercises have a lower risk of developing depression and weak pelvic floor muscles (18). Attitudes toward post-partum physical exercise during postpartum range from 25 to 16% across sub-Saharan Africa (19, 20). Since the women’s postnatal physical fitness was considered “normal,” you can start exercising during the first 4–6 weeks after giving birth, according to numerous experts and organizations, idea including the (ACGO), the U.S. Department of Health and Human Services, and the World Health Organization (5, 17).

The lack of postpartum physical exercise can result in prolonged bed rest for women, leading to sedentary lifestyles. This sedentary behavior may contribute to excessive weight gain, cardiovascular fitness issues, abdominal and pelvic organ prolapse, and overall social disruption. Encouraging and promoting postpartum physical exercise is crucial in mitigating these potential health consequences and supporting women’s overall well-being during the postpartum period (3, 21). To the best of the researcher’s knowledge, there was a notable research gap regarding the practices of postpartum physical exercise among women in the specific study area of Nedjo Town, West Wollega zone, Oromia, Ethiopia. Consequently, this study aimed to address this gap by assessing the practice of postpartum physical exercise and identifying its associated factors among women in the postpartum period in the aforementioned location during the year 2023.

## Methods

### Study design and setting

A community-based cross-sectional study was conducted in Nedjo town, West Wollega zone, Oromia, Ethiopia, from April 20, 2023, to May 20, 2023. Nedjo is a self-administrative town located approximately 515 km west of Addis Ababa. The town consists of five administrative Kebele and has a total population of 45,065, with 49% males and 51% females. The town also has a significant number of children under the age of five, totaling 7,498 (16.64% of the population).

Nedjo town is served by one general hospital, catering to a catchment population of 500,000 to 1,000,000 from six districts. Additionally, there is one urban health center that serves the population of Nedjo town itself. The town is also equipped with four health extension working areas functioning as health posts, along with 11 private medium clinics and 12 drug stores. Both public health facilities and medium clinics in the town provide skilled delivery services and postnatal care for women who have given birth in the last 6 months.

### Source of population

The study included all women of childbearing age residing in Nedjo town as the target population.

### Study population

The study population consisted of all randomly selected women who were in the postpartum period within the last 6 months and were residing in Nedjo town.

### Inclusion criteria

The study included postpartum women aged 18–45 years who had given live birth within the past 6 months and resided in Nedjo town for at least 6 months. Participants who provided informed consent, expressed willingness to participate, and adhered to requirements for postpartum physical exercise assessment were included.

### Exclusion criteria

Women were excluded if they were severely ill, had hearing impairments, experienced severe childbirth complications, or had physical limitations preventing physical activity. Mothers of multiples (e.g., twins or triplets) and women who declined or failed to provide informed consent were also excluded to ensure sample homogeneity.

### Sample size estimation and sampling technique

The required sample size for the study was determined using the single population proportion formula. Assumptions were made based on a proportion of 50% for postpartum physical exercise, considering the absence of previous studies on the topic. A 95% confidence level and a 5% margin of error were considered.

Using the formula *n* = (Zα/2)^2^ * *p* * (1-*p*)/*d*^2^, where:

*n* is the desired sample size,

Zα/2 is the standard normal variable at the desired confidence level (1.96 for a 95% confidence level),

*p* is the proportion (50%),

*q* is the complement of *p* (0.5), and.

*d* is the margin of error (5% or 0.05),

Substituting the values, the calculated sample size was *n* = (1.96)^2^ * 0.5 * 0.5/(0.05)^2^ = 384. After accounting for a 10% non-response rate, the final sample size became *n* = 422.

**Figure 1:**
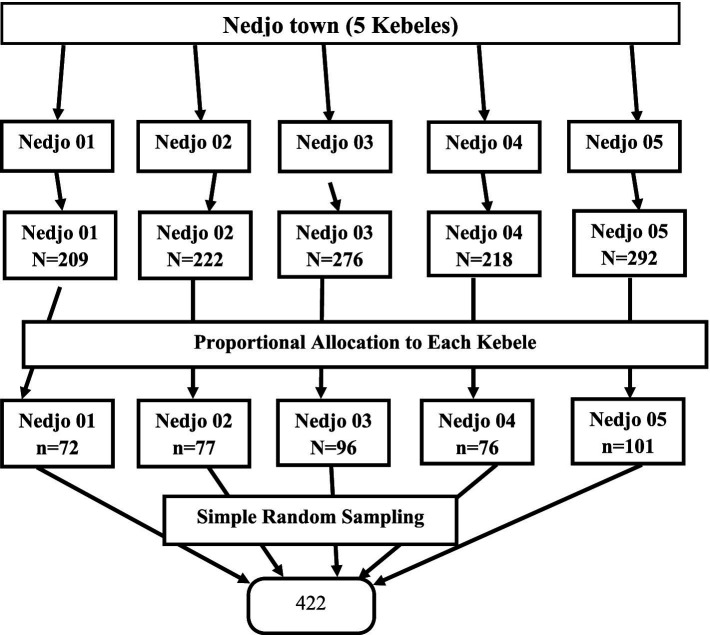
Schematic presentation of sampling procedures of study on assessment of post-partum physical exercise practice and its associated factors among women in the postpartum period in Nedjo town, West Wollega, Oromia, Ethiopia 2023.

A list of postpartum women obtained from the Urban Health Extension Workers registry book for each of the five Kebeles in Nedjo Town was used as the sample frame. Proportionate sample sizes were allocated to each Kebele based on the number of postpartum women registered in the previous 6 months (Figure 1). Simple random sampling was employed to select study participants from the postpartum women registry using a computer random number generator. The selected women were interviewed at their homes, with up to three visits conducted if they were initially missed. In cases where women could not be reached, it was considered a non-response.

### Study variables and measurements

The dependent variable in the study was postpartum Exercise Practice, categorized as either Good or Poor. Postpartum Exercise Practice was defined as engaging in any type of postpartum physical activity during the current postpartum period, as recommended by the American College of Obstetricians and Gynecologists (ACOG) (5).

Good practice: those who exercise any type of postpartum physical exercise in frequency at least 3 times a week and duration ≥20 min per session (5).

Poor practice: exercise any type of postpartum physical exercise in frequency less than 3 times in week and duration <20 min per session (5).

Socio-demographic characteristics: age, religion, educational level, occupation, monthly income, and marital status.

Obstetrical history: gravidity, parity, number of children and history of miscarriage.

Source of information: media, health care provider, family or friends Social support, postpartum (knowledge on postpartum exercise and attitude toward postpartum exercise).

### Data collection instruments and procedures

Data collection was conducted using a structured, interviewer-administered questionnaire. The questionnaire was initially prepared in English and then translated into Afan Oromo by an expert translator. To ensure consistency, the translated version was then back-translated into English by a third person.

Six data collectors, consisting of four BSc nurses and two diploma midwives, were responsible for data collection, with supervision provided by a BSc holder midwife. The questionnaire consisted of two parts: socio-demographic and obstetric history, and a practices assessment tool. The practices assessment tool was adapted from various literature sources (5, 22). Before data collection, the data collectors were provided with an explanation of the study’s objective and its relevance. They were instructed to address any questions or concerns raised by the participants and provide additional clarification as needed. Informed written or non-verbal consent was obtained from each respondent, ensuring their voluntary participation. The data collection process involved using a pre-tested, semi-structured questionnaire in the Afan Oromo language.

### Data quality control

To ensure data quality, several measures were implemented during the study. The questionnaire was carefully designed, standardized, and translated from English to Afan Oromo and back to English to maintain accuracy and consistency. The principal investigator provided a one-day training session for data collectors and supervisors, covering the study’s objectives, confidentiality, data collection procedures, and proper data handling. Guidelines were also given on approaching respondents and addressing any concerns raised.

Before the actual data collection, a pretest was conducted on 5% of the sample size in Gimbi town to assess the validity and reliability of the data collection tool. The internal consistency of the questionnaire was assessed using Cronbach’s Alpha coefficient, which yielded a reliability score of 0.780, indicating a high level of internal consistency for assessing factors associated with postpartum physical exercise practices.

Data collectors and supervisors were introduced to the participants and provided clarification on the questionnaire or any issues that arose during data collection. The supervisors monitored the activities of the data collectors and reviewed all filled questionnaires for completeness, clarity, and proper identification of respondents. Regular checks were performed to ensure the gathered data was complete and presented neatly.

### Data processing and analysis

The collected data were carefully checked for completeness and coded, after which it was entered into EPI data version 4.6 and later transferred to SPSS version 26 for analysis. Descriptive analysis techniques such as frequencies, percentages, and means were applied as necessary. Frequency tables and charts were used to describe categorical variables, while graphs, means, medians, and standard deviations were used for numerical variables.

To identify associations between the outcome and predictor variables, both bivariable and multivariable binary logistic regression analyses were conducted. In the bivariable analysis, independent variables with a *p*-value less than 0.25 were considered for inclusion in the final model. These variables were then subjected to multivariable binary logistic regression to identify significant factors while controlling for confounding effects.

The multivariable analysis provided adjusted odds ratios with their respective 95% confidence intervals, and a *p*-value less than 0.05 was considered statistically significant for identifying significant factors. The Hosmer and Lemeshow’s goodness-of-fit test was performed to assess whether the necessary assumptions for multiple logistic regression were met for all outcome variables. The test yielded a p-value of 0.875 and a chi-square value of 3.682, indicating that the model’s assumptions were fulfilled.

## Results

### Socio-demographic characteristics of the participants

A total of 416 postpartum women participated in this study with a response rate of 98.6%. The mean (±SD) age of the respondent was 26.0 (±4.134) years. The majority of respondents 377 (90.6%) were married. Regarding the educational status of participants, around 145 (34.9%) had a high school education. Concerning the occupation of the participants, 93 (22.4%) were employed. The mean (±SD) average monthly incomes of respondents were 3,951 ± 1997.8 ETB (Table 1).

**Table 1:** Socio-demographic characteristics of postpartum women in Nedjo, West Wollega zone, Oromia Ethiopia, 2023 (*n* = 416).

Variable	Category	Frequency (*n*)	Percent (%)
Age	18–23	99	23.1
	24–29	236	56.7
	30–35	73	17.5
	36–41	11	2.6
Marital status	Single	12	2.9
	Married	377	90.6
	Divorce	18	4.3
	Widowed	5	1.2
Religion	Orthodox	239	57.5
	Protestant	126	30.3
	Muslim	31	7.5
	Catholic	20	4.8
Educational status	No formal education	61	14.6
	Elementary school	131	31.5
	High school	145	34.9
	College and above	79	19
Occupation	Government employed	93	22.4
	Housewife	134	32.2
	Private employed	122	29.3
	Merchant	67	16.1
Income level	<1,500	70	16.8
	1,500–2,999	150	36.1
	3,000–5,999	153	38.2
	6,000–8,999	37	8.9

### Obstetrical characteristics of postpartum women

Majority of the respondents (55.8%) were multipara. About 160 (38.5%) of them were around 6 weeks the postpartum period. More than 4/5th of participants delivered via spontaneous vaginal delivery (Table 2).

**Table 2:** Obstetrical characteristics of postpartum women in Nedjo town West Wollega, Oromia, Ethiopia 2023 (*n* = 416).

Variables	Characteristics	Frequency (*n*)	Percent (%)
Parity	Prim Para	184	44.2
	Multi-para	232	55.8
Weeks after delivery	2 weeks	24	5.8
	3 weeks	41	9.9
	4 weeks	20	4.8
	5 weeks	80	19.2
	6 weeks	160	38.5
	Above 6 weeks	91	21.1
History of miscarriage	Yes	20	5
	No	396	95
Mode of delivery	SVD	336	80.8
	Cesarean section	62	14.9
	AID	18	4.3

SVD, spontaneous vaginal delivery; AID, assisted instrumental delivery.

### Practice of post-partum physical exercise during postpartum among postnatal women

**Figure 2:**
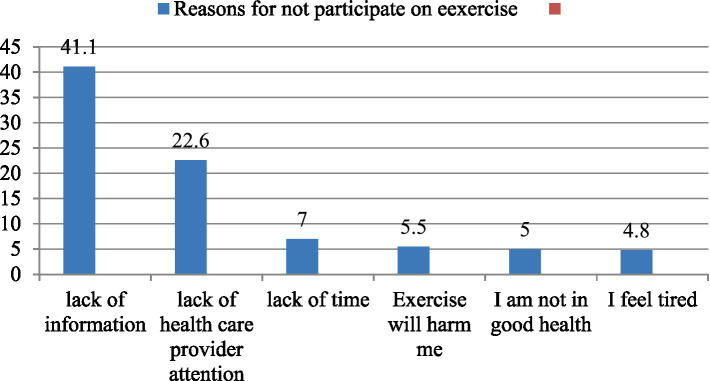
Reasons for not engaged in the current delivery of postpartum physical exercise among Nedjo town, West Wollega, Oromia, Ethiopia, 2023 (*n* = 416).

This study showed that early one-fifth (19.5%) (95% CI: 12.03–22.15) practiced postpartum exercises in the current postpartum period. The main reason reported by participants for not engaging in postpartum physical exercise in the current postnatal period was lack of information (41.1%) (Figure 2).

From those who participated in post-partum physical exercise more than half (53.2%) of them were practicing walking and one-fourth (25%) of them were practicing muscle relaxation exercises. Among those who practiced, 35 (43.2%) of them participants practiced more than three times per week for a duration of greater than 20 min per session, while about 46 (56.8%) of them practiced less than three times per week for a duration of less than 20 min per session of practice.

In addition, among women who were practicing post-partum physical exercise, about 186 nearly half them were informed by the health care provider to practice in the given schedule, while the remaining 142 (34.1%) were advised by family (Table 3).

**Table 3:** Percentage distribution of postpartum physical exercise practice among post-partum women in Nedjo town, West Wollega, Oromia, Ethiopia, 2023.

Characteristic	Frequency (*n*)	Percent (%)
Practicing postpartum physical exercise in the present post-partum period (*n* = 416)		
Yes	81	19.5
No	335	80.5
Type of postpartum physical exercise you exercised now (*n* = 81)		
Walking	43	53.2
Aerobics	20	25
Relaxation and breathing exercises	7	8.2
Pelvic floor exercise	7	8.2
Kegel exercise	6	7.4
Getting information to do postpartum exercise (*n* = 416)		
From health care provider	186	47
From family	142	34.1
Another person (friend)	88	21
Frequency of exercise per week (*n* = 81)		
≤Two times/week	46	56.0
≥ three times/week	35	43.2
Duration of exercise per session (*n* = 81)		
<20 mint	57	70.3
≥20 mint	24	29.6
Perform exercise practice (*n* = 81)		
Poor practice	46	56.8
Good practice	35	43.2

### Associated factors of postpartum physical exercise practice

In multivariable logistic regression, educational level, occupation, parity, Culture, Knowledge of postpartum exercise, and exercise before pregnancy were independently associated with postpartum physical exercise practice.

Women who were government employees were 2 times more likely to practice postpartum physical exercise as compared to housewife women (AOR =2.14; 95% CI: 1.96, 4.74). In addition, women who had completed college and were above the education level were 3 times more likely to practice postpartum physical exercise as compared to those who had no formal education (AOR = 2.97; 95% CI: 1.14, 7.80). Moreover, those prime-para postpartum women were 2.8 times more likely to participate in postpartum physical exercise as compared to those multi-parity women (AOR = 2.81; 95% CI: 1.72, 4.59). Women who had practiced exercise before pregnancy were five times more likely to practice postpartum exercise as compared to those who did not practice before pregnancy [AOR = 5.09, 95% CI: (2.78, 9.29)]. Being knowledgeable on postpartum physical exercise was significantly associated with postpartum physical exercise AOR = 2.30; 95% CI: (1.20, 4.42) (Table 4).

**Table 4:** Bivariable and multivariable logistic regression analysis for the practice of postpartum physical exercise practice among women in the postpartum period in Nedjo town, West Wollega, Oromia, Ethiopia, 2023 (*n* = 416).

Variables	Practice of postpartum exercise *n* (%)		Odd Ratio (95% CI)	AOR 95% CI	*P*-value
	No	Yes	COR(CI)	AOR(CI)	
Educational level					
Non-formal and primary	49(83.1%)	10(16.9%)	1	1	
Primary education	103(92.8%)	8(7.2%)	0.38(0.14,1.02)	1.21(0.18,2.23)	0.234
High school	132(77.6%)	38(22.4%)	1.41(0.65,3.05)	1.32(0.17,2.44)	0.060
College and above	51(67.1%)	25(32.9%)	2.40(1.05,5.52)	2.97(1.14,7.80)*	<0.001
Occupation					
Housewife	98(72.5%)	37(27.4%)	1	1	
Government employee	85(67.5%)	41(32.5%)	1.27(0.16,2.24)	2.14(1.96,4.74)*	<0.001
Private	59(70.2%)	25(29.8%)	1.12(0.21,1.82)	1.2(0.18,2.06)	0.49
Merchant	45(63.4%)	26(36.6%)	1.50(0.45,2.28)	1.10(0.16,1.92)	0.53
Custom (culture) is suited to practice					
No	294(85%)	52(15%)	1	1	
Yes	41(58.6%)	29(41.4%)	3.99(2.29,7.00)	2.7(2.36,5.17)	<0.001
Parity					
Multi parity	230(85.8%)	38(14.2%)	1	1	
Primi parity	105(70.9%)	43(29.1%)	2.48(1.51,4.06)	2.81(1.72,4.59)*	<0.001
Had practice exercise before pregnant					
No	250(88.7%)	32(11%)	1	1	
Yes	87(64.7%)	47(35.1%)	4.22(1.26,6.25)	5.09(2.78,9.29)*	<0.001
Knowledge level					
Poor knowledge	252(89.7%)	30(10.6%)	1	1	
Good Knowledge	88(65.7%)	46(34.3%)	4.36(2.60, 6.59)	2.3(1.20, 4.42)	<0.001

COR, crude odd ratio; CI, confidence interval; AOR, adjusted odd ratio; 1, reference category; *significant at *p* < 0.001.

## Discussion

A community-based cross-sectional study was conducted to assess postpartum physical exercise practices and their associated factors among women in Nedjo Town, West Wollega zone, Oromia, Ethiopia. This study revealed that among those aware of postpartum physical exercise, the types recognized included walking (30.7%), relaxation/stretching (33.8%), aerobics (13.2%), and pelvic floor exercises (10%). These findings align with studies conducted in Nigeria (22) and India (23), where participants similarly identified walking, aerobics, relaxation/breathing exercises, and pelvic floor exercises as forms of postpartum physical activity.

The study found that approximately 81 respondents (19.5%) engaged in postpartum physical exercise during their current postpartum period. This figure is similar to the 22.0% reported in a study from Saudi Arabia (24), but significantly lower than the rates observed in India (58%) (23), Nigeria (84.7%) (22), Canada (29%) (25), and Brazil (29%) (26). The variation in these findings may be attributed to the diverse socio-cultural backgrounds of the study areas, as well as differences in participants’ awareness and understanding of postpartum exercise. Notably, many respondents benefitted from the guidance of healthcare professionals, which contributed to their knowledge of postpartum exercise.

The study demonstrated a statistically significant association between exercising before pregnancy and engaging in postpartum physical activity. This finding aligns with similar research conducted in Cambodia (27) and Canada (25). In contrast, a study from Saudi Arabia reported no significant relationship between pre-pregnancy exercise and postpartum activity among women. This discrepancy may be attributed to variations in socio-cultural behaviors across the study regions (23).

The study’s findings reveal a statistically significant association between government employment and postpartum physical activity among women. Similar results have been observed in studies conducted in South Africa (28), Uganda (11), Vietnam (29), and Sri Lanka (30), underscoring a consistent trend across diverse contexts.

The findings of the current study indicate a statistically significant association between exercising before pregnancy and engaging in physical activity postpartum. These results align with previous research conducted in Brazil (31), West Nigeria (21) and Cambodia (27). Furthermore, this study revealed a notably stronger connection between postpartum exercise behaviors and the support participants received from their families. Similar findings were reported in institutional-based cross-sectional studies from Saudi Arabia (23) and Sri Lanka (30), which highlighted that cultural support for postpartum physical exercise was significantly linked to such activities. The study showed that participants who were their cultural support for postpartum physical exercise was significantly associated with postpartum physical exercise. However, the cross-sectional design may pose temporality issues that could influence the interpretation of cause-and-effect relationships. This study seeks to explore aspects of maternal health that have not been addressed in previous research.

### Strengths and limitations

Self-reported data in this study may introduce biases such as social desirability bias, where participants may provide responses they believe are socially acceptable. This could lead to overestimation or underestimation of certain behaviors, including postpartum physical exercise practices. The cross-sectional design of the study limits the ability to establish causality between variables. It only provides a snapshot of data at a single point in time, making it challenging to determine the temporal sequence of events or establish cause-and-effect relationships. Participants may have difficulty accurately recalling past events or behaviors, such as pre-pregnancy exercise habits, which can introduce recall bias and affect the validity of the results.

## Conclusion

In conclusion, the study revealed that the majority of participants were not engaging in postpartum physical exercise. Women who had completed a college education or higher, were employed in government positions, were primiparous (having one child), had prior exercise experience before pregnancy, and possessed good knowledge about postpartum physical activity were more likely to participate in exercise. The primary reasons cited by women for not exercising included a lack of time, feelings of fatigue and discomfort, and insufficient information.

These findings highlight the urgent need for targeted interventions aimed at postpartum women to raise awareness about the importance of physical exercise during the postpartum period and its associated benefits. Such interventions should specifically address the identified barriers, including time constraints and lack of information, to encourage greater engagement in postpartum physical exercise.

## Data Availability

The original contributions presented in the study are included in the article/supplementary material, further inquiries can be directed to the corresponding author.
